# Novel Bicomponent Functional Fibers with Sheath/Core Configuration Containing Intumescent Flame-Retardants for Textile Applications

**DOI:** 10.3390/ma12193095

**Published:** 2019-09-23

**Authors:** Muhammad Maqsood, Gunnar Seide

**Affiliations:** Aachen Maastricht Institute for Biobased Materials, Faculty of Science and Engineering, Maastricht University, Urmonderbaan 22, 6167 RD Geleen, The Netherlands; muhammad.maqsood@maastrichtuniversity.nl

**Keywords:** bicomponent fibers, intumescent flame-retardants, nonwovens, cone calorimetry

## Abstract

The objective of this study is to examine the effect of intumescent flame-retardants (IFR’s) on the spinnability of sheath/core bicomponent melt-spun fibers, produced from Polylactic acid (PLA) single polymer composites, as IFR’s have not been tested in bicomponent fibers so far. Highly crystalline PLA-containing IFR’s was used in the core component, while an amorphous PLA was tested in the sheath component of melt-spun bicomponent fibers. Ammonium polyphosphate and lignin powder were used as acid, and carbon source, respectively, together with PES as a plasticizing agent in the core component of bicomponent fibers. Multifilament fibers, with sheath/core configurations, were produced on a pilot-scale melt spinning machine, and the changes in fibers mechanical properties and crystallinity were recorded in response to varying process parameters. The crystallinity of the bicomponent fibers was studied by differential scanning calorimetry and thermal stabilities were analyzed by thermogravimetric analysis. Thermally bonded, non-woven fabric samples, from as prepared bicomponent fibers, were produced and their fire properties, such as limiting oxygen index and cone calorimetry values were measured. However, the ignitability of fabric samples was tested by a single-flame source test. Cone calorimetry showed a 46% decline in the heat release rate of nonwovens, produced from FR PLA bicomponent fibers, compared to pure PLA nonwovens. This indicated the development of an intumescent char by leaving a residual mass of 34% relative to the initial mass of the sample. It was found that the IFRs can be melt spun into bicomponent fibers by sheath/core configuration, and the enhanced functionality in the fibers can be achieved with suitable mechanical properties.

## 1. Introduction

The development in process technologies, for the value addition of commercially available polymers, has been steered in the synthetic fiber industry in the recent past [1,2]. Among these technologies, bicomponent melt spinning has established substantial interest in the synthetic fiber industry, due to its prospective applications in the development of numerous innovative fibers, such as ultra-fine fibers, crimped fibers, conductive fibers and fibers with various cross-sectional shapes [3,4]. Bicomponent fibers, with sheath/core (or shell/core) structures, are widely used in the industry for the thermal bonding of nonwoven fabrics [5,6]. Such applications of bicomponent fibers are based on the difference in melting temperatures of the polymers used in the sheath/core structure, e.g., the polymers with lower melting temperatures are employed in the sheath, whereas, high melting temperature polymers are used in the core [7,8]. The objective of bicomponent fibers is to enhance the material’s performance for specific end application. However, the performance of melt-spun bicomponent fibers depend on various factors, particularly on the type of interfaces between both components and behavior of each component in the composition [9,10]. For the superior performance of bicomponent fibers, a stable and uniform interface, with better adhesion of the components, is required [11,12]. Due to the mutual interaction of both the components involved in bicomponent spinning, and the thermal behavior and stress witnessed by each component in spin-line, during extrusion, is considerably different to what they experience in single component melt spinning [13,14]. This difference in their behavior may affect the development of the intended fiber structure. Therefore, the selection of appropriate polymers for each component is necessary for a stable and uniform interface for better adhesion of the components [15,16]. Nowadays, single polymer composites, containing reinforcement and a matrix from the same polymer, are being used in bicomponent melt spinning, in order to have a uniform interface and better interfacial bonding between the components [17,18]. Polylactic acid (PLA) is a synthetic biopolymer, which can be processed into different forms of crystallinity. The amorphous form of PLA can be used as a matrix. Whereas, a highly crystalline form can be used as reinforcement in bicomponent melt spinning process [19,20]. 

Bicomponent spinning is used to impart diverse functionalities in the fibers, such as electrical conductivity, chemical resistance, tactile comfort, and fire resistance [21,22,23]. Hu et al. developed bicomponent fibers with sheath-core and segment-pie configurations for antistatic fabric applications in textiles [24]. Yeom et al. produced spun bonded nonwoven webs from PA6/PE islands-in-the-sea bicomponent fibers for aerosol filtration applications [8]. Yu et al. discussed the radar wave absorbing characterizations of bicomponent fibers with infrared camouflage [22]. The application of bicomponent fibers in fire resistant textiles is a fascinating approach to enhance the performance properties [25]. Fire resistant textiles have diverse applications, such as a firefighter or military apparel, professional racer equipment, beddings, and carpets [26,27]. Therefore, it is critical that fire-resistant or flame retardant textiles. Currently, halogen-containing, flame-retardants (HCFR) are forbidden in the industry. However, exclusions are there for some special applications [28]. The main downside of HCFR is their release of toxic by-products, during combustion, and their harmfulness for the environment and human health. One such solution could be provided by halogen free flame-retardants (HFFR), which are non-toxic alternatives to HCFR, as they do not release toxic by-products during combustion [29]. Intumescent flame retardant (IFR) systems are HFFRs that rely on char formation mechanisms and safe to the environment [30,31]. IFRs usually contain an acid source, a carbon source and a blowing agent. The degradation of the acid source will be catalyzed by heat, resulting in a release of acid, which in turn, dilutes the gas phase and catalyze the dehydration of the carbon source [32]. The resulting char layer creates a barrier between the gas phase and the polymer, reducing heat and mass transfer, thus protecting the material from combustion [33]. Some studies investigated the melt-spinnability of IFR compounds only in single component fibers, but their mechanical properties were too low to be considered for industrial applications [34,35]. In one of our previous research papers [36], we investigated the spinnability of PLA/IFRs composites in mono-component configurations, and found that, although, the multifilament fibers achieved excellent flame retardancy, we only managed to develop bicomponent multifilament fibers with sheath/core configuration. This was carried out to investigate their effect, not only on the fiber’s mechanical properties, but also to test the fire behavior of these novel multifilament fibers. 

Although, various aspects of bicomponent spinning process has been studied in different applications [37,38,39,40,41,42]. However, to the best of our knowledge, no published studies exist to address the effect of the IFRs in bicomponent fibers for textile applications. Therefore, we developed melt spun bicomponent multifilament functional fibers from single polymer, with sheath/core configuration, by using highly crystalline PLA with IFR’s in the core component, while amorphous PLA is in the sheath component. IFR bicomponent fibers were produced on pilot scale melt spinning machine, and the changes in fiber mechanical properties and crystallinity were recorded in response to varying process parameters. We also tested the thermal stability of bicomponent fibers by thermogravimetric analysis, and the additive dispersion was analyzed by scanning electron microscopy. Thermally bonded nonwoven fabric samples from as prepared bicomponent fibers were produced and their fire properties, such as limiting oxygen index and cone calorimetry data, such as, heat release rate, time to ignition and residual mass% were measured. While, the ignitability of fabric samples were tested by single-flame source test.

## 2. Materials and Methods 

Two different types of PLA pellets, i.e., highly crystalline PLA (Luminy L130) and amorphous PLA (Luminy LX930) were purchased from Total-Corbion (Gorinchem, Netherlands). The physical properties of both types of PLA are presented in Table 1. Halogen free flame retardant (Exolit AP 422) having decomposition temperature higher than 275 °C was attained from Clariant (Muttenz, Switzerland). Exolit AP 422 is a fine-particle ammonium polyphosphate (APP) containing 31% (w/w) phosphorous and 14% (w/w) nitrogen, having density of 1.9 g·cm^−3^ and average particle size of 17 μm, used as acid donor in intumescent formulations. The kraft lignin (KL) powder “UPM BioPiva 100” was purchased from UPM Biochemicals (Helsinki, Finland). A modified polyester based plasticizing agent (PES) in white granular form (thermally stable up to 280 °C) was obtained from Preluna (Ludwigshafen, Germany), to improve spinnability of IFR composites. PLA (Luminy L130), APP, PES and KL were vacuum dried at 100 °C for 4 h before compounding.

Coperion ZSK Mc^18^ twin-screw extruder (Stuttgart, Germany) was used to prepare PLA/APP/PES/KL composites for the core component of bicomponent fibers. In the first feeding zone, highly crystalline PLA pellets (Luminy L130) were mixed together with plasticizing agent (PES) whereas in the second feeding zone, flame retardant (APP) was mixed with kraft lignin powder (KL). PLA/APP/PES/KL composites were prepared at screw rotation speed of 500 rpm to use as core component for bi-component fibers. The melt flow index (MFI) of the blends were in the range of 26–30 g/10 min. The formulations, with content of each component (w/w), as prepared composites are presented in Table 2. The temperatures of the three heating zones were kept at 170 °C, 175 °C, and 180 °C, respectively. 

**Figure 1:**
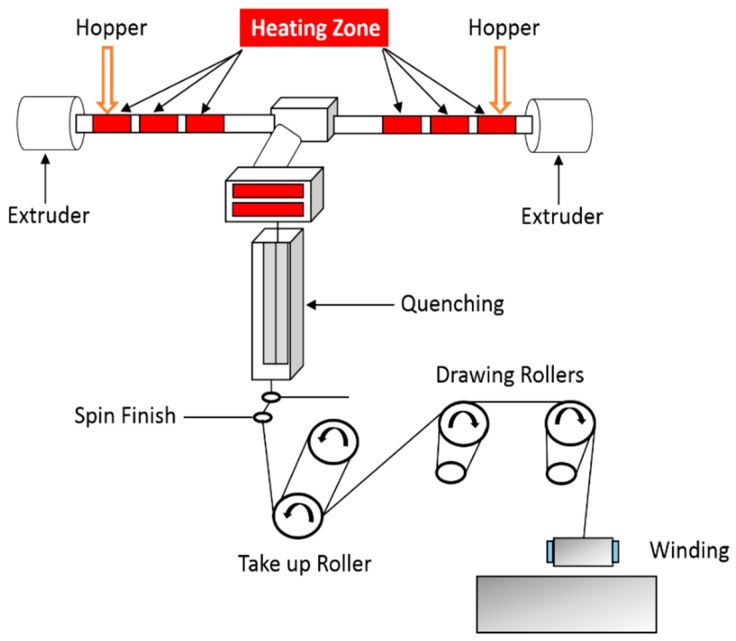
Schematic diagram of pilot scale bicomponent melt spinning machine.

PLA/APP/PES/KL pellets to be used as the core component were vacuum dried at 100 °C for 4 h, whereas, PLA Luminy LX930 pellets, used as sheath components, were vacuum dried at 40 °C for 24 h before spinning, as per the instructions of the suppliers. Vacuum drying of the polymers is necessary to avoid possible filament breakage during melt spinning, due to lowered viscosity of the melt caused by the hydrolysis caused by excessive moisture in the polymers. The moisture content of the pellets was lower than 75 ppm, as determined by Karl Fischer Titrations method, according to ASTM D6869-03 [43]. The bicomponent fibers were melt spun using Fourne Maschinenbau GmbH (Impekoven, Germany) pilot scale melt spinning machine, which have the capacity to produce bicomponent fibers, consisting of two different polymers, with a throughput of up to few kilograms per hour. A schematic diagram of the bicomponent plant is shown in Figure 1. PLA/APP/PES/KL pellets (core component) were fed into one hopper and PLA Luminy LX930 pellets (sheath component) were dosed in the second hopper of bicomponent melt spinning machine. Pellets were melted at a temperature range of 195 °C to 220 °C by using a separate extruder for each type of pellet. The coaxially-combined melted material was then injected into a spinneret plate with each hole for bicomponent monofilaments of sheath/core configuration. Spin pumps, which rotated at constant revolutions per minute (rpm), ensured a homogeneous supply of the material to the spinneret plate. The spin pump for each component delivered a fixed quantity of the melt to the specially designed spinneret for the bicomponent fibers. Through the different rpm of the spin pumps for sheath and core, the fixed quantity of the melt for each component was delivered, and in this way, the thickness of each component was adjusted.

The monofilaments were passed through quenching section (quenching length = 1.4 m) where they were cooled down by maintaining the cool air velocity of 0.5 m·s^−1^ and then combined together to multifilaments, by applying a spin finish before they were collected by the suction gun. The multifilaments were passed through a take up roller followed by hot drawing at two set of heated rollers rotating at varying speeds. The multifilaments with sheath/core configuration were then wound on bobbins rotating on a winder for further analysis. The extruding sheath/core ratio for all bicomponent fibers was set to 1:2 (thin sheath and thick core) under constant melt flow.

To further investigate the spinnability of bicomponent functional fibers in response to varying process parameters, a design of experiment was prepared using half factorial design in MINITAB 18 statistical software (Minitab LLC, State College, PA, USA). Four factors/variables with two levels each were considered in this study and are shown in Table 3. Spinning parameters such as solid state draw ratio (SSDR), temperature (°C) of the heating rollers, linear density (dtex) of multifilaments and spinneret type with different numbers of filaments were varied, in order to analyze the effect of these parameters on the mechanical, structural, and thermal properties of the bicomponent fibers. It is important to mention here that SSDR is the solid-state draw ratio. This means that fibers are drawn when they are solidified. It is different from the term melt draw ratio (MDR). In MDR, filaments are drawn from the spinneret plate until the take up roller by varying its speed, whereas in SSDR, filaments are drawn from the take up roller until the last pair of drawing rollers on melt spinning machine.

The design of experiment showing different spinning parameters used to produce bicomponent fibers is presented in Table 4. Two spinneret types consisting of 24 and 34 number of filaments, two SSDR of 1.2 and 1.4, two temperature conditions 50 °C and 70 °C of the heating rollers and two different linear densities 400 dtex and 600 dtex of multifilaments were selected to investigate the effect of these parameters on fibers properties. 

The mechanical properties of bicomponent fibers, such as tenacity (cN·tex^−1^) and elongation at break (%), were tested on Zwick Roell testing machine by using the EN ISO 5079 standard method [44]. The specimen lengths (50 mm) and rate of deformation (50 mm·min^−1^) were kept constant for all samples. Ten specimens were prepared from each sample and their average results with standard deviations were recorded.

Thermogravimetric analysis of bicomponent fibers was done using a TGA Q5000 device (TA Instruments, New Castle, DE, USA). The specimens (15–20 mg) were heated at a constant rate of 10 °C·min^−1^ up to 700 °C under nitrogen at a flow rate of 50 mL·min^−1^. The thermal decomposition temperature, and the temperature at which maximum degradation took place, were calculated along with the residual mass percentage of the sample compared with the initial mass. The thermogravimetric curves of specimens were plotted after analysis.

The surface morphology, cross sectional images and additives dispersion in bicomponent fibers were evaluated by using a Hitachi S-3200 scanning electron microscope (Chiyoda, Tokyo, Japan). For better cross-sectional images, the bicomponent fibers were first immersed in liquid nitrogen to be freezed and then were cut with a razor blade. Standard specimen stubs with silver coating were used to attach the samples, which were then sputter coated with gold. Selective samples were inspected at a magnification of 1000 × and at an accelerating voltage of 20 kV. 

Differential scanning calorimetry (DSC) analysis of bicomponent fibers was done using a DSC Q2000 (TA instruments). Nitrogen gas was used at a stream rate of 50 mL·min^−1^. The samples were heated at a constant rate of 10 °C·min^−1^ starting from 0 °C to 230 °C, and then cooled at the same rate. The degree of crystallinity (Xc) of bicomponent fibers was calculated by Equation (1),

(1) $$\mathrm{Xc}\;\%=\frac{\Delta \mathrm{Hm}-\Delta \mathrm{Hcc}\;}{\Delta \mathrm{Hf}\;\times \mathrm{Wfr}\;}\;\times 100$$

where Xc corresponds to degree of crystallinity of the sample; ΔHm implies to heat of fusion of the sample; ΔHcc corresponds to cold crystallization enthalpy; ΔHf relates to the heat of fusion of 100% crystalline material, and Wfr is the net weight fraction of the polymer. The heat of fusion of 100% crystalline PLA (ΔHf) is approximately 93.6 J·g^−1^.

**Figure 2:**
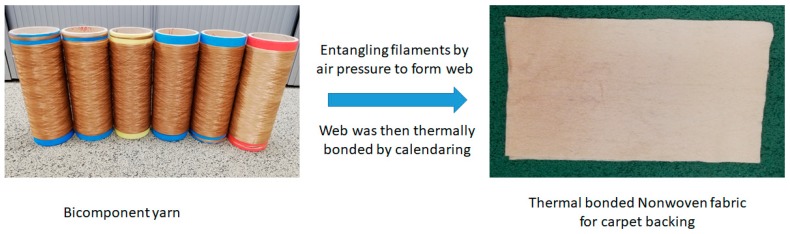
Bicomponent filaments and thermal bonded nonwoven fabric.

Thermally bonded nonwoven fabrics were produced from flame retardant bicomponent fibers to be used as carpet backing. The process comprised the steps in laying down the continuous bicomponent filaments and entangling them together at filament crossover points by air pressure, which then were laid on the conveyer belt to form a nonwoven web. The nonwoven web was thermally bonded by calendaring, at a temperature of 135 °C, which is the melting temperature of the sheath material (low melting PLA) in the bicomponent yarn. After bonding the web thermally by passing over a rotating roller heated by a hot air; the web was passed through a cooling section to consolidate the fixation and then wound up on fabric rolls. The resultant nonwoven flame retardant fabric was dimensionally stable and had a width of around 50 cm as shown in Figure 2. The nonwoven fabric produced had an areal density of 120 g·m^−2^. 

The limiting oxygen index (LOI) of samples was tested. LOI is the fraction of oxygen that must be present to support burning, hence higher LOI values indicate lower flammability. The nonwoven samples (100 × 10 × 3 mm^3^, as required by ISO 4589-2 [45]) were vertically placed in a glass column supplied with a mixture of oxygen and nitrogen gas, and were then ignited from above using a downward-pointing flame. The LOI tests for nonwoven samples were conducted using the Fire Testing Technology (FTT) Oxygen Index Apparatus (Stanton Redcroft, Sussex, UK).

Thermally bonded nonwoven fabric samples with dimensions of 100 × 100 × 3 mm^3^ were prepared to perform a cone calorimetry test, according to ISO 5660 [46], with heat flux of 35 kW·m^−2^ by using Stanton Redcroft instrument (Illinois Toolworks, Glenview, IL, USA). The cone calorimeter equipment operates on an oxygen consumption principle and measures the total heat of combustion, based on the amount of oxygen consumed. Peak heat release rate (PHRR), time to ignition (TTI), and residual mass (%) of thermally bonded nonwoven fabric samples, produced from FR bicomponent fibers, were recorded after the test. Cone calorimetry testing was also performed on pure PLA nonwoven fabric sample for the comparison purpose. 

The single-flame source test (ignitability test) as required by EN ISO 11925-2 [47] was conducted by using FTT Ignitability Apparatus (FTT, Sussex, UK). In this fire test, nonwoven fabric samples of size 250 mm × 90 mm were exposed to a direct gas flame of a height of 20 mm after placing them vertically on a U-shaped sample holder. The flame application point was 40 mm above the bottom surface of the fabric sample. To observe the falling flame droplets, a filter paper was also positioned underneath the sample holder. The flame application time was set for 15 s for each test and after 20 s of removal of flame the test was terminated. After terminating the test, flame spread distance (mm) and burning time was measured. The fabric classification was based on whether the flame spread reached 150 mm distance in a given time period, or whether the filter paper positioned below the sample holder was ignited by the flame droplets or not. 

## 3. Results and Discussion

### 3.1. Melt Spinning and Mechanical Properties of Bicomponent Fibers

The spinning of bicomponent multifilament fibers was not only challenging, but it also took a while to find the right parameters to make the spinning process stable for continuous collection of fibers. The melt spinning process was stable without filament breakage for as spun fibers or for the fibers drawn at lower solid-state draw ratio (SSDR). However, an occasional filament breakage were observed when drawn at higher SSDR. This suggests that bicomponent fibers containing IFRs in the core component can be melt spun in to multifilaments if not drawn at too high SSDR. In our case, SSDR up to 1.4 was sufficient to produce bicomponent fibers without filament breakage. However, beyond this SSDR, filaments kept on breaking and continuous collection of the fibers was not possible. IFRs were used in the core component and not in the sheath component of the bicomponent fibers because previous studies [38,40] suggests that the components with higher viscosities should be placed in the core and not in the sheath. The reason is that, in coaxial flow, the sheath component is driven through higher stress areas than core component. If additives are used in the sheath component its melt viscosity will be increased further, and will not be able to withstand the tensions and stresses during processing hence, stable process will not be built-up. 

**Figure 3:**
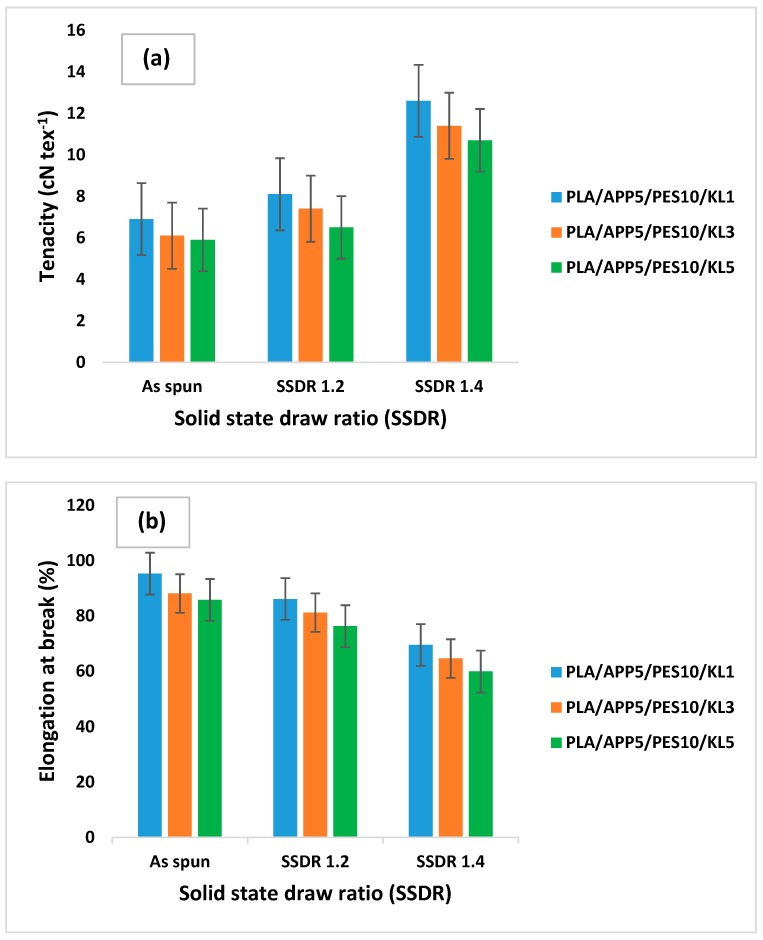
Tenacity (**a**) and elongation at break (**b**) of PLA/APP/PES/KL bicomponent fibers.

The summary of the test results are presented in Table 5, while the mechanical properties of the melt-spun bicomponent fibers are shown in Figure 3. The tenacity of the as spun fibers containing 1 wt % of KL were on the lower side (6.9 cN·tex^−1^) as expected, since no crystallinity was induced in the fibers. However, it was significantly improved by increasing SSDR as a maximum tenacity of 12.6 cN·tex^−1^ containing 1 wt % KL was achieved at SSDR of 1.4. The tenacity was gradually reduced by increasing the wt % of KL in the core component at the same SSDR of 1.4. It can be seen in Table 5 that the addition of 3 wt % and 5 wt % of KL, in the core of bicomponent fibers, could only achieve a tenacity of 11.4, and 10.7 cN·tex^−1^ respectively at the same SSDR of 1.4. Compared to as spun fibers, the fibers drawn at SSDR of 1.2, showed lower elongation at break (%), while the increasing SSDR of 1.4, the elongation at break further reduced, as shown in Table 5 and Figure 3. These results indicated that the mechanical properties of melt spun bicomponent fibers were not only dependent of SSDR but, loading concentration (%) of KL also played a significant role in defining the mechanical properties of bicomponent fibers. It is noteworthy to mention that, amongst all factors, only SSDR was found to be the most significant factor that affected the mechanical properties of fibers. Other factors such as, number of filaments, drawing roller temperatures and yarn linear density hardly affected mechanical or thermal properties of the fibers. Therefore, their effect on fiber properties has not been mentioned here. 

Similar findings were reported by Hu et al. [24] and observed that a dramatic down fall in mechanical properties of core/sheath bicomponent fibers, containing antistatic agents occurred by increasing filler content, and suggested that a strong interaction between matrix and filler content is required in order to improve mechanical properties of composite fibers. Lund et al. [48] used carbon black as conductive core in poly (vinylidene fluoride) (PVDF) matrix and found that mechanical properties of fibers decreased quite significantly at any loading concentration of carbon black compared to neat PVDF fibers, even though the fillers were uniformly distributed within the matrix.

### 3.2. Thermogravimetric Analysis 

**Figure 4:**
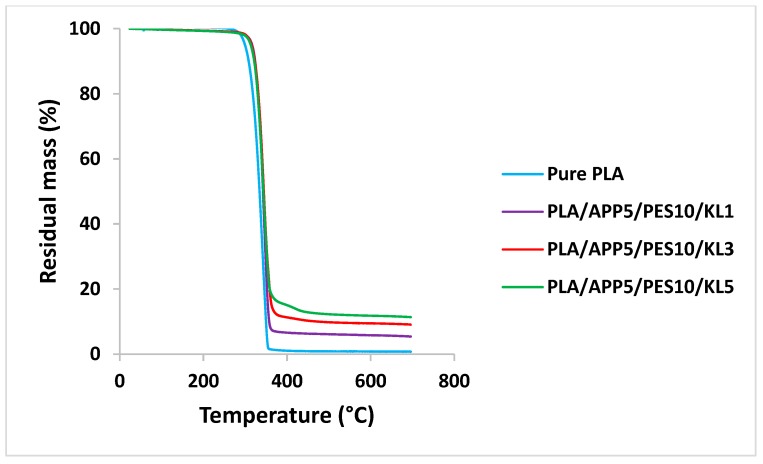
Thermogravimetric curves of bicomponent fibers.

The effect of IFR additives on thermal stability and thermal decomposition of bicomponent fibers was investigated by thermogravimetric analysis and the residual mass percentage of samples was determined at 700 °C. The thermal decomposition temperatures and mass residue percentage of the samples after TG analysis were compared to determine the influence of carbonization agent (KL) on thermal stabilities of bicomponent composite fibers. TG curves of bicomponent fibers and their respective data for the samples heated in a nitrogen atmosphere are presented in Figure 4, and Table 6, respectively.

The temperatures corresponding to 5% and 50% mass loss for the bicomponent fibers are represented by T5 and T50 values in Table 6. Whereas, the temperature corresponding to the maximum rate of mass losses is represented by T max. The degradation of the bicomponent fiber containing 1 wt % of KL (PLA/APP5/PES10/KL1) started at 315.2 °C and 50% loss occurred at 343.3 °C, with residual mass left at 700 °C of 5.3%. The thermal stabilities of the bicomponent fiber containing 3 wt % of KL (PLA/APP5/PES10/KL3) were slightly higher to that of composite fiber containing 1 wt % of KL however, the residue left at 700 °C was 9.0% of the initial mass of the sample. For the bicomponent fiber containing 5 wt % of KL (PLA/APP5/PES10/KL5), the initial decomposition temperatures and thermal stabilities were greater than the corresponding values for the bicomponent fibers containing 1 and 3 wt % of KL, with 11.3% residual mass left at 700 °C. No residual mass was left for pure PLA fibers and their T5 and T50 values were also lower compared to fibers containing KL. The addition of higher wt % of lignin (3 and 5%) not only improved the thermal stability of the composite fibers, but also increased the residual mass% at 700 °C. For example, the bicomponent fiber containing 3 wt % of KL (PLA/APP5/PES10/KL3) increased the residual mass to 9.0%, compared to 5.3% of composite fiber containing 1 wt% of KL. The addition of 5 wt % of KL (PLA/APP5/PES10/KL5) further increased the residual mass to 11.3% at 700 °C. 

The thermogravimetric curves of bicomponent fibers containing 1, 3, and 5 wt % of KL are presented in Figure 4. The bicomponent fiber, containing 5 wt % of KL, was found to be more thermally stable and presented denser and more compact char structure with higher residual mass (11.3%), due to charring ability of KL as a result of polycyclic aromatic hydrocarbons formation as indicated by Sharma et al. [49]. Thermogravimetric curves presented in Figure 4 of bicomponent fibers show the residual mass percentage as a function of temperature, up to 700 °C. The reason for the selection of only as spun fibers for TGA analysis is that, SSDR did not had any effect on the thermal stability or on residual mass (%) of fibers. The parameter that affects most the thermal stability of fibers was the loading concentration of the additives incorporated in the polymer blends. Therefore, we only selected the as spun fibers with different loading concentration of KL for TGA analysis.

The curves indicate that most of the thermal decomposition occurs between 300 °C and 400 °C. Whereas, all the bicomponent fibers decompose within a narrow temperature window, thereby, increasing the concentration of KL causes more residual mass to remain at temperatures between 375 °C and 700 °C. The thermal stabilities of bicomponent fibers, containing 5 wt % of KL are, therefore, better than those bicomponent fibers containing 1 and 3 wt % of KL. This behavior is mainly due to higher decomposition temperatures of these fibers for 5% and 50% mass loss (T5 and T50 values) than other fibers. The other reasons could also be due to the greater char-forming ability of the polyhydric component (KL) and dehydration mechanism, established by acid source (APP). As the formation of phosphate compounds that further enhance the dehydration of KL result in higher char formation with compact structures.

### 3.3. Scanning Electron Microscopy

**Figure 5:**
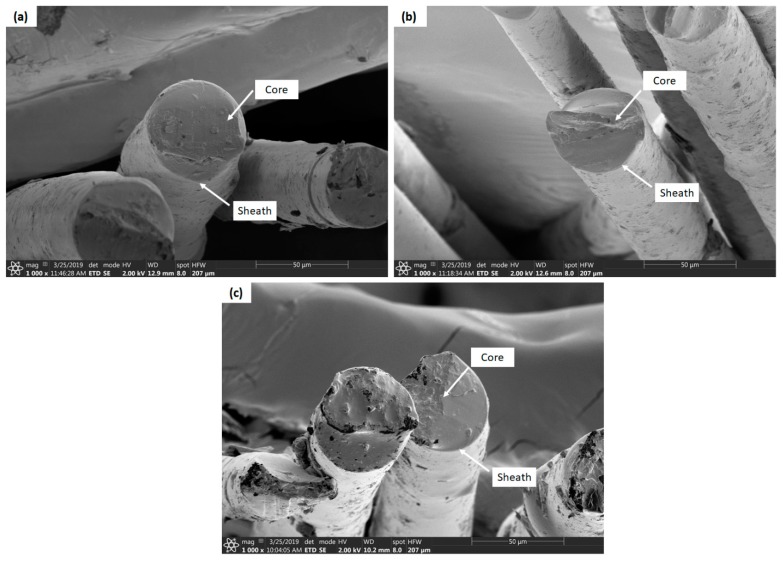
Cross-sectional SEM images of PLA/APP5/PES10/KL1 (**a**), PLA/APP5/PES10/KL3 (**b**), PLA/APP5/PES10/KL5 (**c**), bicomponent composite fibers [1000 × and 50 μm].

**Figure 6:**
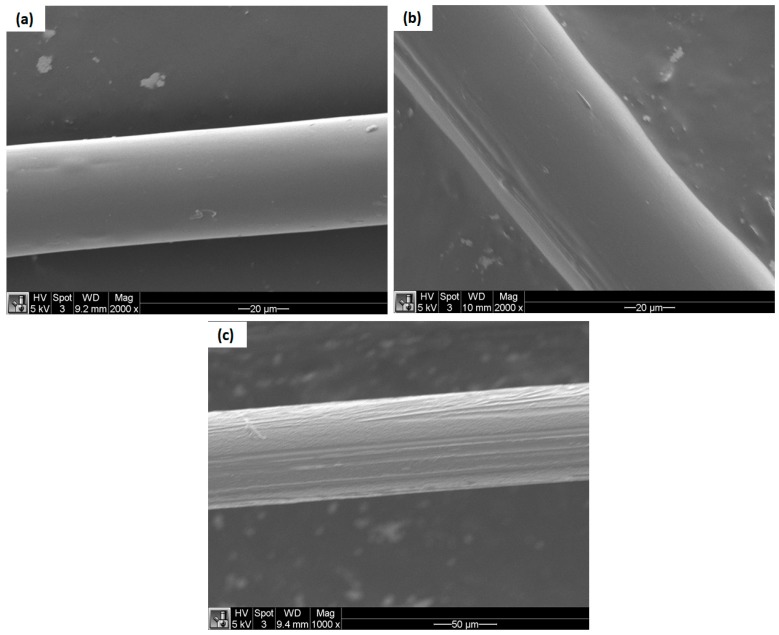
Surface SEM images of PLA/APP5/PES10/KL1 (**a**), PLA/APP5/PES10/KL3 (**b**), PLA/APP5/PES10/KL5 (**c**), bicomponent composite fibers [1000 × and 20 μm].

The dispersion characteristics of IFR additives and the microstructures of bicomponent fibers were examined by scanning electron microscopy. The cross-sectional images and surface morphology of bicomponent fibers are shown in Figure 5 and Figure 6, respectively. It can be seen by SEM images that the dispersion state of IFR additives is uniform in the bicomponent fibers. SEM images showed some exfoliated and tactoid regions, which developed from the large aggregates of the additives that had accumulated at the fiber surface. However, due to a strong shear rate and consistent elongational stress, applied at higher draw ratios during melt spinning, a significant intercalation was seen and a more uniform dispersion of IFR additives were observed. In the longitudinal direction of bicomponent fibers, a stripe was observed as shown in Figure 6b,c which was developed from thermal shrinkage of the core component of the fibers. It occurred as a result of the crystallization (solidification) of the sheath component at much lower temperatures to the core component, as similar effects were observed by Houis et al. [25] and Kazemi et al. [37]. As the crystallized sheath component had a much lower coefficient of thermal expansion than the polymer melt in the core component, a significant volume reduction occured with the cooling of the polymer melt in the core component of the fibers. This volume reduction led to the stripe development in the longitudinal direction of the fibers, as similar behaviour of the core/sheath components was reported by Ayad et al. [10]. Since the core-sheath configuration we used had a higher volume flow rate for the core, a faster cooling rate had to be applied in the quenching section to maintain balance. Due to the faster cooling rate in the quenching section, the filaments from the outside (sheath) solidified much earlier than from the inner side (core) and, as a consequence, shrinkage of the core material took place during congealing.

### 3.4. Differential Scanning Calorimetry

**Figure 7:**
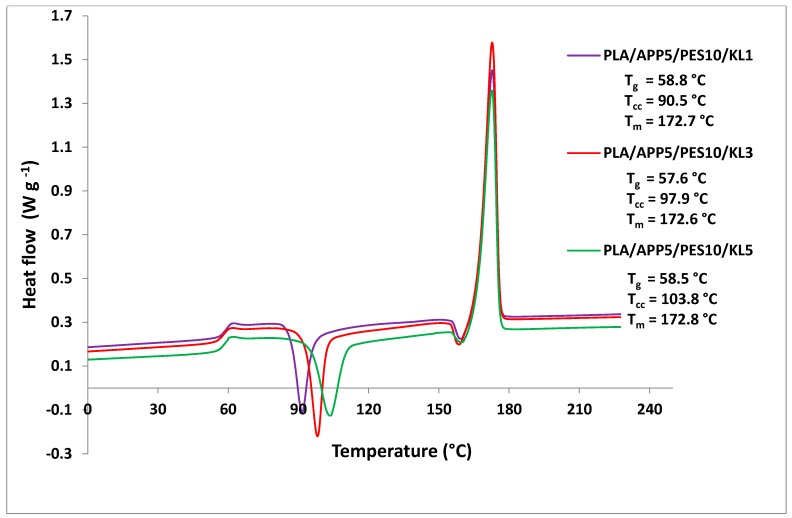
Differential scanning calorimetry (DSC) thermograms of bicomponent fibers.

Differential scanning calorimetry was used to investigate the crystallinity of the bicomponent fibers. DSC curves of the bicomponent fibers are presented in Figure 7. 

**Figure 8:**
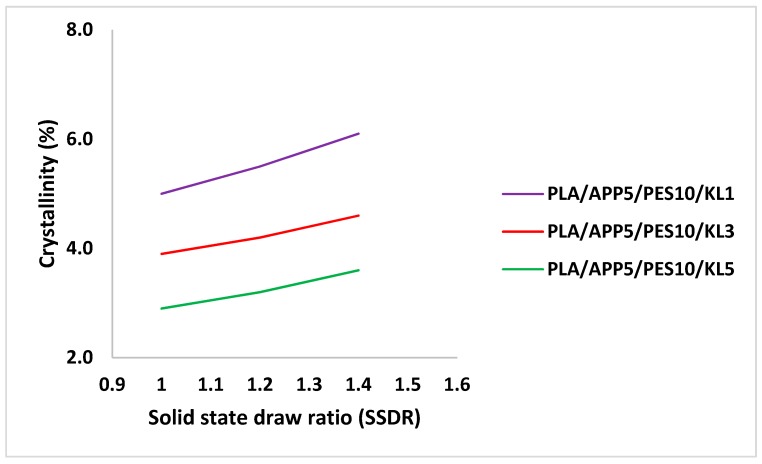
Crystallinity of bicomponent fibers as a function of draw ratio.

The peaks for the glass transition temperatures, cold crystallization temperatures, and melting temperatures can be clearly seen for all the three-bicomponent fibers in the DSC thermograms. However, all the three bicomponent fibers have different cold crystallization exotherms, which is attributed to the differently oriented and semi-crystallized core and sheath components. The thermal properties and crystallinity (%) of the bicomponent fibers, calculated by Equation (1), are presented in Table 7. Whereas, the crystallinity (%) of the bicomponent fibers, as a function of the draw ratio, is shown in Figure 8. It was observed that crystallinity of the bicomponent fibers increased by increasing the SSDR, which is due to a better alignment of the molecular chain within the polymers.

In melt spinning, the crystallinity of fibers changed by hot drawing them (stretching between two heated rollers). This attenuated and aligned the molecular chain within the fibers and, by doing so, their crystallinity increased. The bicomponent fibers containing 1, 3, and 5 wt % of KL in the core component (PLA/APP5/PES10/KL1, PLA/APP5/PES10/KL3 and PLA/APP5/PES10/KL5) showed glass transition temperatures at 58.8 °C, 57.6 °C, and 58.5 °C, respectively, while, melting peaks were at 172.7 °C, 172.6 °C, and 172.8 °C, respectively. However, a major difference in their cold crystallization peaks was seen. 

Table 7 demonstrates that the bicomponent fibers, containing 5 wt % of KL (PLA/APP5/PES10/KL5), presented a cold crystallization peak at higher temperature (103.8 °C). Whereas, the bicomponent fibers, containing 1 wt % of KL (PLA/APP5/PES10/KL1), showed cold crystallization peak at much lower temperatures (90.5 °C). This is because a higher concentration of KL (5 wt %) increased the viscosity of the melt, more agglomerates were formed and less uniform dispersion of additives took place. Therefore, the cold crystallization temperature increased due to the restriction in polymer chain mobility. In DSC, the thermograms of all three bicomponent fibers, resulted in an exothermic peak before the melting peak was observed at around 158 °C. 

Table 7 demonstrates that the bicomponent fiber, containing 1 wt % of KL, have the highest crystallinity (6.1%) than the rest of the bicomponent fibers. The reason for that is that the bicomponent fiber, with at least wt% of the additives, spent enough time in the processing apparatus for cooling which enabled it to form all possible crystallites. In contrast, the bicomponent fibers with higher wt % of additives in the core took longer to crystallize as the fast cooling in the processing apparatus did not enable them to form all possible crystallites, and their crystallinity was reduced. Therefore, it presented less time for the orientation of the macromolecules in the cooling section, which led to partial crystallinity of the core component and the overall crystallinity of the fiber was reduced. Another factor which influenced the crystallization behavior of the polymers is the heat transfer from core to the sheath component, and then to the air. The greater the heat transfer from the core to the sheath component, the higher the crystallinity of the fibers [50].

### 3.5. Fire Testing

Cone calorimetry gives useful insights on the burning behavior of materials by calculating parameters, such as peak heat release rate (PHRR), time to ignition (TTI), and residual mass (%) after burning of the material. The cone calorimetry data of pure PLA nonwoven fabric sample and nonwoven fabric samples, produced from FR PLA bicomponent fibers, are presented in Table 8. The influence of three different wt % of KL (i.e., 1, 3 and 5 wt %) in nonwoven fabric samples were assessed against their reactions to fire. TTI of pure PLA nonwoven fabric sample was 73.2 s. TTI of the fabric sample containing 1 wt % of KL (PLA/APP5/PES10/KL1) was 62.4 s, which was decreased to 59.3 s in case of 3 wt % of KL (PLA/APP5/PES10/KL3) and to 57.1 s when 5 wt % of KL was incorporated in the fabric sample. 

When a material is decomposed, pyrolysis gases are released, and if the concentration of such gases is reached at certain threshold, the ignition of the material starts. Prolonged ignition time of a material is mainly due to slower decomposition of the material. The samples containing KL showed lower TTI than pure PLA, as it fits with common intumescent systems as the degradation of the intumescent samples has to begin prior charred layer is developed.

**Figure 9:**
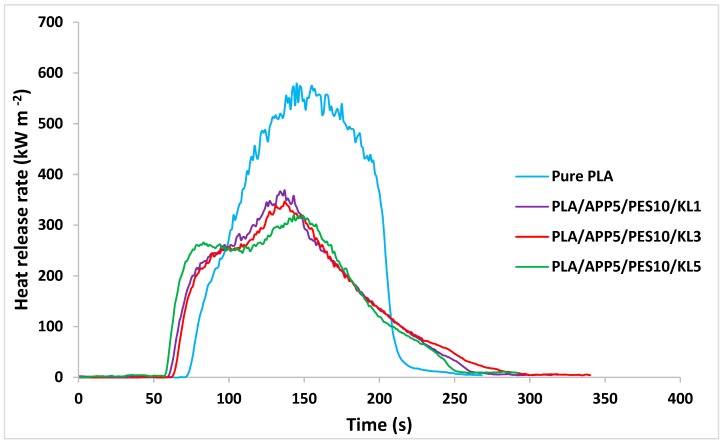
Heat release curves of pure PLA and FR PLA nonwoven fabric samples.

The curves showing peak heat release rate (PHRR) of the fabric samples after cone calorimetry are presented in Figure 9. Pure PLA nonwoven sample burnt considerably quicker than the other nonwoven samples and produced a very steep PHRR curve with a PHRR of 573.6 kW·m^−2^. The fabric sample containing 1 wt % of KL after ignition presented PHRR of 368.2 kW·m^−2^. For the fabric sample containing 3 wt % of KL, PHRR declined to 337.1 kW·m^−2^, which was further reduced to 309.3 kW m^−2^ by the addition of 5 wt % of KL. These findings suggested that higher loading (%) of KL formed thicker and compact char layer on fabric surface which restricted further burning of the sample and helped in extinguishing the fire. The residual mass (%) of nonwoven fabric samples after cone calorimetry are also presented in Table 8. There was no residual mass left for pure PLA nonwoven sample after cone calorimetry test. The residual mass left for fabric sample containing 1 wt % of KL was 21.3% which was increased to 26.7% and 34.5% by the addition of 3 wt %, and 5 wt % of KL, respectively. The higher residual mass (%) in case of fabric sample containing 5 wt % of KL reflected an increased char production, due to lower PHRR values. 

**Figure 10:**
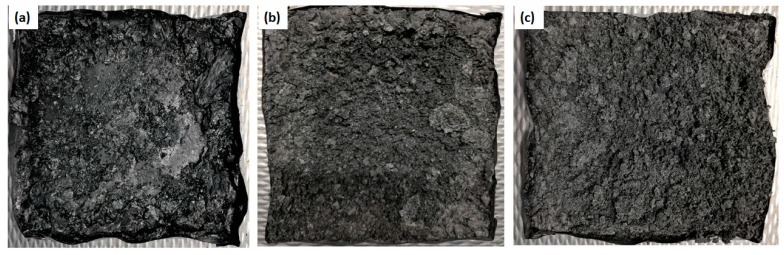
Char residues of fabric samples (**a**) 1% KL (**b**) 3% KL (**c**) 5% KL after cone calorimetry.

The intumescent system operates in the condensed phase where the burning of the material generates sponge-like multicellular structure (char), which protects the underlying material from further heat and mass transfer by acting as a physical barrier between the material and source of fire. Figure 10 showed the char residues of the fabric samples after cone calorimetry test. 

The fabric samples containing 1 wt % of KL presented a thinner and porous char layer due to decreased viscosity of the substrate a very little pressure was built-up, which allowed gas bubbles and vapors to escape from the unclosed cells, resulting in reduced swelling of the char layer and increased heat release rate. On the other hand, more compact and uniform char presented by the sample, containing 5 wt % of KL, prevented the free escape of gas bubbles and vapor particles. This resulted in greater pressure build-up, due to closed cells, which further increased the melt viscosity of the condensed substrate hence, greater swelling of char can be seen. 

The limiting oxygen index (LOI) test is widely accepted to assess the flame retardancy of materials. We also tested the LOI values of nonwoven fabric samples, which are presented in Table 8. The LOI value of fabric sample containing 1 wt % of KL was 25.2%. The addition of 3 wt % of KL increased the LOI value to 27.1% and the presence of 5 wt % of KL further enhanced the LOI value to 30.4%. Higher LOI values for the samples containing greater content% of KL is mainly due to the formation of char layer, which not only protected the fabric sample from external heat source, but also protected it from further degradation. 

The ignitability test under EN ISO 11925-2 method was used to determine the ignitability of nonwoven fabric samples in the vertical direction by direct flame impingement. Six specimens from each fabric sample were subjected to surface exposure to flame and their results are presented in Table 9. Six specimens from each fabric sample were exposed to flame for 15 (s) and their flame spread time (s) and flame spread distance (mm) were measured. It can be seen in Table 9 that none of the fabric sample containing KL was ignited after 15 (s) of flame exposure, and therefore, flame spread distance (mm) and time (s) could not be recorded. Neither the burning droplets from the specimen could be seen nor was the ignition of the filter paper placed underneath of the specimen.

**Figure 11:**
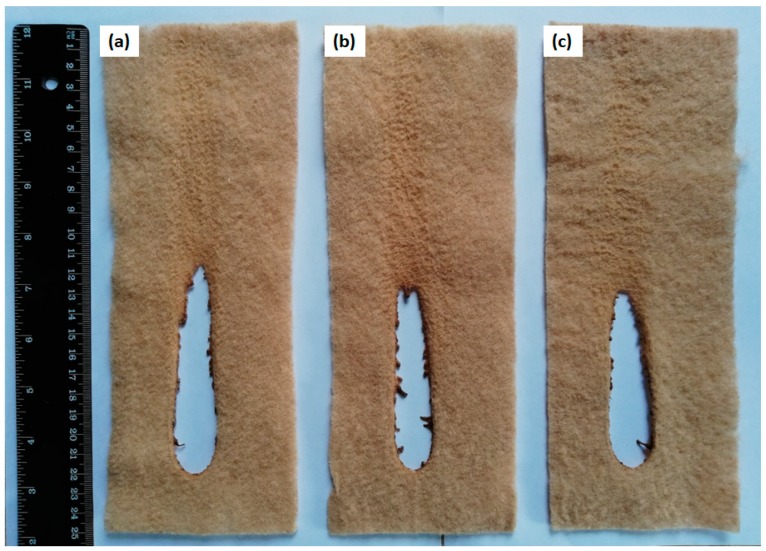
Images of PLA/APP5/PES10/KL1 (**a**), PLA/APP5/PES10/KL3 (**b**), PLA/APP5/PES10/KL5 (**c**) FR nonwoven fabric samples after ignitability test.

The reason for no ignition of the specimen was an increase in the gap between flame and the specimen, due to the fleeing of heat flux. The temperature at the specimen surface decreased and heat flow was reduced hence, fuel availability was no more to continue ignition. Based on this test our fabric samples got the classification of E and Efl. To get E and Efl (for floorings) classification the flame should not reach the top marking (150 mm) within 20 s after 15 s of flame exposure and there should be no burning droplets and no ignition of the filter paper placed underneath. It can be seen in Figure 11 that flame could not reach the distance of 150 mm after 15 s of flame exposure. 

## 4. Conclusions

In this study, novel bicomponent functional fibers, based on single polymers, consisting of highly-crystalline PLA with intumescent flame retardants in the core and low melting PLA were produced in the sheath. Thermogravimetric analysis confirmed that the thermal stabilities of bicomponent fibers increased quite significantly, and the fibers containing 5 wt % of KL presented the highest residual mass%. Thermally bonded nonwoven fabric samples produced from bicomponent fibers showed remarkable flame retardancy. A significantly low PHRR (309.3 kW·m^−2^) of sample containing 5 wt % of KL was observed, which is 46% less than the PHRR of the nonwoven sample, which was made from pure PLA. TTI of KL samples decreased quite significantly since fabric sample containing 5 wt % of KL presented TTI of 57.1 (s) compared to 73.2 (s) for pure PLA, which fits best with common intumescent systems as degradation of the intumescent samples has to begin before the charred layer is developed. The nonwoven fabric sample, containing 5 wt % of KL, showed a residual mass of up to 34.5% relative to the initial mass of the sample, thereby indicating the development of an intumescent char. This protects the fabric sample from further burning. Limiting oxygen index of the fabric samples was also tested and the highest LOI (30.4%) was observed for the sample containing 5 wt % of KL. The ignitability test showed none of the fabric sample, produced from bicomponent fibers, was ignited after 15 (s) of flame exposure, and therefore, achieved classification of E and Efl as per the standards set for EN ISO 11925-2 method. This certifies that the product can be used commercially for floor coverings. Compared to our previously published PLA/IFR mono-component multifilament fibers with tenacity of up to 7.3 cN·tex^−1^, the tenacity of the bicomponent fibers was improved up to 12.6 cN·tex^−1^. Hence it was confirmed that IFRs can be melt spun into bicomponent fibers by sheath/core configuration, while flame retardant nonwoven fabrics can be produced for FR applications.

## Figures and Tables

**Table 1:** Physical properties of Polylactic acid (PLA) pellets.

Physical Properties	Unit	PLA Luminy L130	PLA Luminy LX930
Density	g/cm^3^	1.24	1.24
Melt flow index	g/10 min	24	17
Stereochemical purity	%	≥ 99 (L-isomer)	90 (L-isomer)
Appearance	Visual	Crystalline white pellets	Amorphous white pellets
Melting temperature	°C	175–180	125–135
Glass transition temperature	°C	55–60	55–60

**Table 2:** Additives composition in PLA composites.

No.	Formulations	PLA (wt %)	APP (wt %)	PES (wt %)	KL (wt %)
1	PLA/APP5/PES10/KL1	84	5	10	1
2	PLA/APP5/PES10/KL3	82	5	10	3
3	PLA/APP5/PES10/KL5	80	5	10	5

**Table 3:** Factors and their levels.

No.	Factors	Levels	
1	No of filaments	24	34
2	Solid state draw ratio (SSDR)	1.2	1.4
3	Temperature of draw rollers (°C)	50	70
4	Linear density (dtex)	400	600

**Table 4:** Design of the experiment.

Sample	No. of Filaments	Solid State Draw Ratio (SSDR)	Temperature of Draw Rollers (°C)	Yarn Linear Density (dtex)
PLA/APP5/PES10/KL1	24	1.2	50	400
	34	1.2	50	600
	24	1.4	50	600
	34	1.4	50	400
	24	1.2	70	600
	34	1.2	70	400
	24	1.4	70	400
	34	1.4	70	600
PLA/APP5/PES10/KL3	24	1.2	50	400
	34	1.2	50	600
	24	1.4	50	600
	34	1.4	50	400
	24	1.2	70	600
	34	1.2	70	400
	24	1.4	70	400
	34	1.4	70	600
PLA/APP5/PES10/KL5	24	1.2	50	400
	34	1.2	50	600
	24	1.4	50	600
	34	1.4	50	400
	24	1.2	70	600
	34	1.2	70	400
	24	1.4	70	400
	34	1.4	70	600

**Table 5:** Summary of mechanical properties of bicomponent melt-spun fibers.

Formulation	SSDR	Tenacity (cN·tex^−1^)	Elongation at Break (%)
PLA/APP5/PES10/KL1	As spun	6.9 ± 1.3	95.3 ± 6.4
	SSDR 1.2	8.1 ± 1.6	86.1 ± 9.7
	SSDR 1.4	12.6 ± 1.1	69.5 ± 11.3
PLA/APP5/PES10/KL3	As spun	6.1 ± 1.5	88.1 ± 10.5
	SSDR 1.2	7.4 ± 1.9	81.2 ± 11.1
	SSDR 1.4	11.4 ± 1.6	64.6 ± 13.5
PLA/APP5/PES10/KL5	As spun	5.9 ± 1.2	85.8 ± 10.9
	SSDR 1.2	6.5 ± 1.7	76.3 ± 16.6
	SSDR 1.4	10.7 ± 1.9	59.9 ± 12.8

**Table 6:** Data of thermogravimetric analysis.

No.	Formulations	T5 (°C)	T50 (°C)	T Max (°C)	Residual Mass (%)
1	Pure PLA	300.3 ± 1.7	334.6 ± 1.3	340.1 ± 1.8	0.0 ± 0.0
2	PLA/APP5/PES10/KL1	315.2 ± 2.3	343.3 ± 1.9	356.4 ± 2.9	5.3 ± 1.6
3	PLA/APP5/PES10/KL3	317.8 ± 3.1	347.9 ± 2.6	359.2 ± 2.1	9.0 ± 1.8
4	PLA/APP5/PES10/KL5	321.4 ± 2.9	351.6 ± 3.6	363.7 ± 1.9	11.3 ± 1.1

**Table 7:** Thermal properties of bicomponent fibers by DSC analysis.

No.	Samples	Tg (°C)	Tcc (°C)	Tm (°C)	∆Hm (J·g^−1^)	∆Hcc (J·g^−1^)	Xc (%)
1	PLA/APP5/PES10/KL1	58.8 ± 0.6	90.5 ± 0.8	172.7 ± 0.9	34.2 ± 0.3	29.4 ± 0.4	6.1 ± 0.7
2	PLA/APP5/PES10/KL3	57.6 ± 0.4	97.9 ± 0.6	172.6 ± 1.1	30.9 ± 0.5	27.3 ± 0.3	4.6 ± 0.3
3	PLA/APP5/PES10/KL5	58.5 ± 0.5	103.8 ± 0.7	172.8 ± 1.3	27.6 ± 0.4	24.9 ± 0.5	3.6 ± 0.8

Tg = glass transition temperature, Tcc = cold crystallization temperature, Tm = melting temperature, ∆Hm = heat of fusion of sample, ∆Hcc = cold crystallization enthalpy, Xc = crystallinity of fibers.

**Table 8:** Cone calorimetry data for pure PLA and FR PLA nonwoven fabric samples.

No.	Formulation	TTI (s)	PHRR (kW m^−2^)	THR (MJ m^−2^)	Residual Mass (%)	LOI (%)
1	Pure PLA	73.2 ± 1.4	573.6 ± 12.1	58.1± 0.4	0.0 ± 0.0	19.3 ± 1.9
2	PLA/APP5/PES10/KL1	62.4 ± 1.3	368.2 ± 7.4	38.4 ± 0.2	21.3 ± 0.4	25.2 ± 1.2
3	PLA/APP5/PES10/KL3	59.3 ± 1.7	337.1 ± 4.8	35.8 ± 0.9	26.7 ± 0.6	27.1 ± 0.9
4	PLA/APP5/PES10/KL5	57.1 ± 1.3	309.3 ± 6.9	33.1 ± 0.4	34.5 ± 0.5	30.4 ± 1.6

TTI = time to ignition; HHR = heat release rate; THR = total heat release; LOI = limiting oxygen index.

**Table 9:** Ignitability test results of nonwoven fabric samples.

No.	Formulation	Ignition (Yes/No)	Time for Flame Tip to Reach 150 mm (s)	Extent of Flame Spread (mm)	Burning Droplets	Ignition of Filter Paper
1	PLA/APP5/PES10/KL1	No	Did not reach	None	No	No
2	PLA/APP5/PES10/KL3	No	Did not reach	None	No	No
3	PLA/APP5/PES10/KL5	No	Did not reach	None	No	No

## References

[1] Prahsarn C., Klinsukhon W., Padee S., Suwannamek N. (2016). Hollow segmented-pie PLA/PBS and PLA/PP bicomponent fibers: an investigation on fiber properties and splittability. J. Mater. Sci..

[2] Oh T.H. (2006). Effects of Spinning and Drawing Conditions on the Crimp Contraction of Side-by-Side Poly (trimethylene terephthalate) Bicomponent Fibers. J. Appl. Polym. Sci..

[3] Kikutani T., Radhakrishnan J., Arikawa S., Takaku A., OKUI N., Jin X., Niwa F., Kudo Y. (1996). High-speed Melt Spinning of Bicomponent Fibers: Mechanism of Fiber Structure Development in Poly (ethy1ene terephthalate)/ Polypropylene System. J. Appl. Polym. Sci..

[4] Zhang D., Sun C., Beard J., Brown H., Carson I.A.N., Hwo C. (2002). Development and Characterization of Poly (trimethylene terephthalate) -Based Bicomponent Meltblown Nonwovens. J. Appl. Polym. Sci..

[5] Godshall D., White C., Wilkes G.L. (2001). Effect of Compatibilizer Molecular Weight and Maleic Anhydride Content on Interfacial Adhesion of Polypropylene–PA6 Bicomponent Fibers. J. Appl. Polym. Sci..

[6] Yeom B., Pourdeyhimi B. (2011). Web fabrication and characterization of unique winged shaped, area-enhanced fibers via a bicomponent spunbond process. J. Mater. Sci..

[7] Durany A., Anantharamaiah N., Pourdeyhimi B. (2009). High surface area nonwovens via fibrillating spunbonded nonwovens comprising Islands-in-the-Sea bicomponent filaments: structure– process–property relationships. J. Mater. Sci..

[8] Yeom B., Pourdeyhimi B. (2011). Aerosol filtration properties of PA6/ PE islands-in-the-sea bicomponent spunbond web fibrillated by high-pressure water jets. J. Mater. Sci..

[9] Dasdemir M., Maze B., Anantharamaiah N., Pourdeyhimi B. (2012). Influence of polymer type, composition, and interface on the structural and mechanical properties of core/sheath type bicomponent nonwoven fibers. J. Mater. Sci..

[10] Ayad E., Rault A., Gonthier A., Campagne C., Devaux E. (2018). Effect of Viscosity Ratio of Two Immiscible Polymers on Morphology in Bicomponent Melt Spinning Fibers. Adv. Polym. Technol..

[11] Dasdemir M., Maze B., Anantharamaiah N., Pourdeyhimi B. (2011). Formation of novel thermoplastic composites using bicomponent nonwovens as a precursor. J. Mater. Sci..

[12] Oh T.H. (2006). Melt Spinning and Drawing Process of PET Side-by-Side Bicomponent Fibers. J. Appl. Polym. Sci..

[13] Choi Y.B., Kim S.Y. (1999). Effects of Interface on the Dynamic Mechanical Properties of PET/Nylon 6 Bicomponent Fibers. J. Appl. Polym. Sci..

[14] Sun C.Q., Zhang D., Liu Y., Xiao R. (2004). Preliminary Study on Fiber Splitting of Bicomponent Meltblown Fibers. J. Appl. Polym. Sci..

[15] Cho H.H., Kim K.H., Kang Y.A., Ito H., Kikutani T. (1999). Fine Structure and Physical Properties of Polyethylene/Poly (ethylene terephthalate) Bicomponent Fibers in High-Speed Spinning. I. Polyethylene Sheath/ Poly (ethylene terephthalate) Core Fibers. J. Appl. Polym. Sci..

[16] Zhao R.R., Wadsworth L.C. (2003). Study of Polypropylene/Poly (ethylene terephthalate) Bicomponent Melt-Blowing Process: The Fiber Temperature and Elongational Viscosity Profiles of the Spinline. J. Appl. Polym. Sci..

[17] Naeimirad M., Zadhoush A., Kotek R., Khorasani S.N., Ramakrishna S. (2018). Recent advances in core/shell bicomponent fibers and nanofibers: A review. J. Appl. Polym. Sci..

[18] Zhao R.O.N.R., Wadsworth L.C., Zhang D., Sun C. (2002). Polymer Distribution During Bicomponent Melt Blowing of Poly (propylene)/Poly (ethylene terephthalate). J. Appl. Polym. Sci..

[19] Maqsood M., Seide G. (2018). Statistical modeling of thermal properties of biobased compostable gloves developed from sustainable polymer. Fibers Polym..

[20] Maqsood M., Seide G. (2018). Development of biobased socks from sustainable polymer and statistical modeling of their thermo-physiological properties. J. Clean Prod..

[21] Cho H.H., Kim K.H., Kang Y.A., Ito H., Kikutani T. (1999). Fine Structure and Physical Properties of Poly (ethylene terephthalate)/Polyethylene Bicomponent Fibers in High-Speed Spinning. II. Poly (ethylene terephthalate) Sheath/ Polyethylene Core Fibers. J. Appl. Polym. Sci..

[22] Yu B., Qi L., Ye J., Sun H. (2006). Preparation and Radar Wave Absorbing Characterization of Bicomponent Fibers with Infrared Camouflage. J. Appl. Polym. Sci..

[23] Wang X.Y., Gong R.H. (2005). Thermally Bonded Nonwoven Filters Composed of Bicomponent Polypropylene/Polyester Fiber. I. Statistical Approach for Minimizing the Pore Size. J. Appl. Polym. Sci..

[24] Hu C., Chang S., Liang N. (2018). Fabrication of antistatic fibers with core/sheath and segmented-pie configurations. J. Ind. Text..

[25] Houis S., Schmid M., Lubben J. (2007). New Functional Bicomponent Fibers with Core/Sheath-Configuration Using Poly (phenylene sulfide) and Poly (ethylene terephthalate). J. Appl. Polym. Sci..

[26] Avinc O., Day R., Carr C., Wilding M. (2012). Effect of combined flame retardant, liquid repellent and softener finishes on poly(lactic acid) (PLA) fabric performance. Text. Res. J..

[27] Cheng X.W., Guan J.P., Tang R.C., Liu K.Q. (2016). Improvement of flame retardancy of poly(lactic acid) nonwoven fabric with a phosphorus- containing flame retardant. J. Ind. Text..

[28] Karim M.N., Rigout M., Yeates S.G., Carr C. (2014). Surface chemical analysis of the effect of curing conditions on the properties of thermally-cured pigment printed poly (lactic acid) fabrics. Dyes Pigm..

[29] Bourbigot S., Duquesne S., Fontaine G., Bellayer S., Turf T., Samyn F. (2008). Characterization and Reaction to Fire of Polymer Nanocomposites with and without Conventional Flame Retardants. Mol. Cryst. Liq. Cryst..

[30] Maqsood M., Seide G. (2018). Investigation of the Flammability and Thermal Stability of Halogen-Free Intumescent System in Biopolymer Composites Containing Biobased Carbonization Agent and Mechanism of Their Char Formation. Polymers.

[31] Gordobil O., Delucis R., Egüés I., Labidi J. (2015). Kraft lignin as filler in PLA to improve ductility and thermal properties. Ind. Crops Prod..

[32] Maqsood M., Langensiepen F., Seide G. (2019). The Efficiency of Biobased Carbonization Agent and Intumescent Flame Retardant on Flame Retardancy of Biopolymer Composites and Investigation of their melt spinnability. Molecules.

[33] Costes L., Laoutid F., Aguedo M., Richel A., Brohez S., Delvosalle C., Dubois P. (2016). Phosphorus and nitrogen derivatization as efficient route for improvement of lignin flame retardant action in PLA. Eur. Polym. J..

[34] Cayla A., Rault F., Giraud S., Salaün F., Fierro V., Celzard A. (2016). PLA with intumescent system containing lignin and ammonium polyphosphate for flame retardant textile. Polymers.

[35] Solarski S., Mahjoubi F., Ferreira M., Devaux E., Bachelet P., Bourbigot S., Delobel R., Coszach P., Murariu M., Da silva Ferreira A. (2007). (Plasticized) Polylactide/clay nanocomposite textile: Thermal, mechanical, shrinkage and fire properties. J. Mater. Sci..

[36] Maqsood M., Langensiepen F., Seide G. (2019). Investigation of melt spinnability of plasticized polylactic acid biocomposites-containing intumescent flame retardant. J. Therm. Anal. Calorim..

[37] Kazemi S., Mojtahedi M., Takarada W., Kikutani T. (2014). Morphology and Crystallization Behavior of Nylon 6-Clay/Neat Nylon 6 Bicomponent Nanocomposite Fibers. J. Appl. Polym. Sci..

[38] Lund A., Jonasson C., Johansson C., Haagensen D., Hagstr B. (2012). Piezoelectric Polymeric Bicomponent Fibers Produced by Melt Spinning. J. Appl. Polym. Sci..

[39] Liu Y., Cheng B., Wang N., Kang W., Zhang W., Xing K., Yang W. (2012). Development and Performance Study of Polypropylene/Polyester Bicomponent Melt-Blowns for Filtration. J. Appl. Polym. Sci..

[40] Straat M., Rigdahl M., Hagstrom B. (2011). Conducting Bicomponent Fibers Obtained by Melt Spinning of PA6 and Polyolefins Containing High Amounts of Carbonaceous Fillers. J. Appl. Polym. Sci..

[41] Ding Z., Qi L. (2008). Preparation of Sheath–Core Bicomponent Composite Ion-Exchange Fibers and Their Properties. J. Appl. Polym. Sci..

[42] Wang X.Y., Gong R.H. (2006). Thermal Oxidative Degradation of Bicomponent PP/PET Fiber During Thermal Bonding Process. J. Appl. Polym. Sci..

[43] (2003). ASTM D6869-03: Standard Test Method for Coulometric and Volumetric Determination of Moisture in Plastics Using the Karl Fischer Reaction (the Reaction of Iodine with Water).

[44] (1995). EN ISO 5079: Textile fibres—Determination of breaking force and elongation at break of individual fibres.

[45] (2017). ISO 4589-2: Plastics—Determination of burning behaviour by oxygen index—Part 2: Ambient-temperature test.

[46] (2015). ISO 5660-1: Reaction-to-fire tests—Heat release, smoke production and mass loss rate—Part 1: Heat release rate (cone calorimeter method) and smoke production rate (dynamic measurement).

[47] (2010). ISO 11925-2: Reaction to fire tests—Ignitability of products subjected to direct impingement of flame—Part 2: Single-flame source test.

[48] Lund A., Hagstro B. (2011). Melt Spinning of b -Phase Poly (vinylidene fluoride) Yarns With and Without a Conductive Core. J. Appl. Polym. Sci..

[49] Sharma R.K., Wooten J.B., Baliga V.L., Lin X., Chan W.G., Hajaligol M.R. (2004). Characterization of chars from pyrolysis of lignin. Fuel.

[50] Rwei S., Jue Z., Chen F.L., Laboratories U.C. (2004). PBT/PET Conjugated Fibers: Melt Spinning, Fiber Properties and Thermal Bonding. Polym. Eng. Sci..

